# Structure and Properties of Polyamide Fabrics with Insect-Repellent Functionality by Electrospinning and Oxygen Plasma-Treated Surface Coating

**DOI:** 10.3390/polym12102196

**Published:** 2020-09-25

**Authors:** Chunhui Xiang, Nicholas R. Etrick, Margaret W. Frey, Edmund J. Norris, Joel R. Coats

**Affiliations:** 1 Department of Apparel, Events, and Hospitality Management, Iowa State University, Ames, IA 50011, USA; 2 Department of Materials Science & Engineering, University of Michigan, Ann Arbor, MI 48109, USA; netrick@umich.edu; 3 Department of Fiber Science & Apparel Design, Cornell University, Ithaca, NY 14853, USA; 4 Department of Entomology and Nematology, University of Florida, Gainesville, FL 32611, USA; ej.norris@ufl.edu; 5 Department of Entomology, Iowa State University, Ames, IA 50011, USA; jcoats@iastate.edu

**Keywords:** polyamide, insect repellency, electrospinning, plasma-treated, dip coating, permethrin, efficacy

## Abstract

The need for light-weight and high-strength insect-repellant fabrics is of critical importance to the cessation of viral diseases. The goal of the study is to investigate the structure and properties of insect-repellent polyamide fabrics for use in protective garments to guard against mosquitos. Permethrin was applied to the polyamide fabrics through incorporation into the nylon 6 polymer solution during electrospinning and dip coating onto the control untreated and oxygen plasma-treated polyamide fabrics: electropun nylon 6 nanofiber nonwovens, commercially available nylon 6 warp knit tricot, and nylon 66 double weft, knit interlock fabrics. The incorporation of permethrin into the polymer solution before the formation of fibers demonstrated the most efficient way to apply permethrin to the fiber/fabric systems. The plasma treatment significantly increased the amount of permethrin on the surface of the fabrics. All permethrin-containing polyamide fabrics showed excellent fastness of the insecticide to light. The electrospun nylon 6 nonwovens demonstrated the best fastness to washing among the plasma-treated electrospun nylon 6, nylon 66 double weft knit, and nylon 6 warp-knit tricot. All permethrin-treated fabrics were repellent and caused higher percentage of mosquito escape compared to the control untreated fabrics.

## 1. Introduction

The increasing temperatures across the globe have given rise to the spread of Zika and other insect-driven viruses by means of dissemination through mosquitoes [1]. This phenomenon is driving a market need for a product that is able to not only prevent insect bites, but also reduce the effects of the spread of these insects. Personal protective measures against biting arthropods and arthropod-borne diseases constitute the first line of defense [2]. A major advance in the protection of high-risk personnel (e.g., outdoor workers, travelers, and soldiers) has been the development of topical repellent formulations and residual insecticides that can be impregnated into clothing, tents, and netting [3,4]. Currently, there exist numerous commercial products available on the market with the insecticide permethrin. Permethrin (3-phenoxybenzyl [1RS]-cis, trans-3-[2, 2-dichlorovinyl]-2,2-dimethylcyclopropanecarboxylate), which belongs to the synthetic pyrethroids, is one commonly used agent and has applications as a common insecticide, acaricide, and repellent agent with a broad range in its activity spectra [5]. Permethrin combines the essential qualities of repellency, hot-feet, knockdown, kill, and residual activity and has been widely used for decades as an arthropod-contact repellent in fabric impregnation [6]. Permethrin is currently the only insecticide allowed by the Environmental Protection agency (EPA) for usage in clothing fabrics, due to its low toxicity to humans and negligible effects from leaching onto skin from contact [7]. As a personal protective additive, permethrin is used as a repellent in cloth impregnation and has applications primarily for the treatment of military uniforms and mosquito nets [8]. Permethrin is a chiral compound with two stereocenters in the cyclopropane ring and is usually produced as a mixture of the two stereoisomers (cis/trans ratios of 40:60, 80:20 or 25:75) [9]. The EPA, in 2009, revised their exposure and risk assessment of repellent-treated clothing and reported that permethrin factory-treated clothing is unlikely to pose any significant immediate or long-term hazard to people wearing the clothing [10]. Various brands, such as L.L. Bean, E.X. Officio, REI, and Insect Shield, offer variations of permethrin-treated clothing. However, these clothing items are often thick, heavy, expensive, and non-disposable. Therefore, the search for an alternative method for fabricating light-weight, high-strength, and disposable permethrin-treated fabrics is of utmost importance.

Polyamides (nylon 6 and nylon 66) are the most widely used polyamides for fibers and engineering materials [11]. The properties of polyamides, which include high strength ability, abrasion resistance, and resilience, make them very important in the manufacture of clothing and carpets. Polyamide 6, also known as nylon 6, is an aliphatic polyamide characterized by recurring amide groups (–CONH–) in the polymeric chain and amino and carboxylic end groups [12]. Nylon 6 has a rapid crystallization rate and has been shown to produce strong electrospun fibers. Nylon 6 nanofibers produced by electrospinning exhibit excellent mechanical properties, such as toughness and high tensile strength [8,9,10,11,12]. 

Electrospinning is the most widely used approach in producing nanofiber membranes. Increasing customer demand for durable and functional apparel manufactured in a sustainable manner has created an opportunity for nanomaterials to be integrated into textile substrates [13]. The electrospun fibers can be collected as a randomly laid nonwoven fabric with an exceptionally large surface-to-mass ratio, high porosity, and small pore size. The small diameter of electrospun fibers leads to large, specific surface areas in electrospun fabrics. This large surface area has been shown to provide increased absorbency over other textile fabrics [14]. The high surface area and small pore sizes have provided a significant advantage to the electrospun fabrics over knit and mesh fabrics that have large pore sizes with less viability for application on and incorporation into the insecticide on the surface.

Plasma treatment of organic materials is a technique employed to modify their surfaces and enhance properties such as the following: adhesive bonding, durability, wettability, bio-compatibility, and adhesion of dyes [12]. The impact is profound at a shallow depth of the polymer surface, leaving the bulk practically unaffected. At the same time, the plasma glow discharge assists in the removal of a weak boundary layer (WBL) residing on the surface, serving as a cleaning process as well. Plasma treatment is an alternative method to wet chemical treatments, provides a uniform modification, is not environmentally hazardous, and requires short process times. Pippa et al. [12] modified the surfaces of polyamide fibers and films using atmospheric plasmas. In their study, nylon fibers and films were plasma-treated with nitrogen, helium, and acetylene under atmospheric pressure conditions. Their results revealed an improvement of the wettability of the surfaces accompanied by an increase in their surface tension. The modified materials showed an alteration in their surface composition, which results mainly from the oxygen uptake. The modified surfaces are rougher and, therefore, are ideal for advanced composite systems that require enhanced adhesive properties.

An area of particular interest in insect-repellent textile fabrication and design is the protection of human beings, animals, and the textile material itself against insect attack [6]. Different methods for impregnation of fabrics with repellents have been previously reported: dipping [15], spraying [16,17,18], polymer-coating [2,6], metal–organic framework (MOF) deposition on fibers/fabrics [19], and microencapsulation methods [20,21]. Most of the listed techniques exploit pyrethroids as active substances. For example, Abdelhammeed et al. [19] reported that a Ti-bearing metal–organic framework (MOF) supported on cellulosic fabrics (cotton, viscous, and linen) had excellent antimosquito properties, attracting and killing the insects. In their modified fabrics, the covalent bonding between the MOF and the silica-modified fabrics contributed to good washing resistance, surviving more than five washing cycles. Abdel-Mohdy et al. studied the repellency of controlled release-treated cotton fabrics based on permethrin and bioalletrin [22], and cypermethrin and prallethrin [23], against mosquitoes. The finished cotton fabrics in their study showed fast repellent action, slower knockdown action, and instant killing action. However, limited work has been done on investigating the effect of fabric structures and the methods of incorporating active ingredients on insect repellent efficacy. Furthermore, little work has been done on incorporating active ingredients into electrospun nanofibers in personal protective clothing. 

In this study, insect-repellent polyamide fabrics were developed by incorporating permethrin into nylon 6 fibers during electrospinning, and dip coating permethrin onto as-spun/received and oxygen-plasma-treated electropun nylon 6, commercially available nylon 6 warp-knit tricot and nylon 6, 6 double weft-knit interlock fabrics. Oxygen plasma treatment was used to modify the polyamide fabric surfaces to enhance the bonding of permethrin with the fabrics. The polyamide fabrics were characterized by structural properties, surface chemistry, pore size, and distribution. The permethrin wet pickup efficiencies of control untreated and plasma-treated polyamide fabrics were compared. The permethrin-treated polyamide fabrics were also investigated with regard to the fastness of the insecticide during washing or to light. Contact Irritancy Assay (CIA) was used to evaluate the repellent efficacy of the permethrin-containing polyamide fabrics.

## 2. Materials and Methods

### 2.1. Materials

Nylon 6 (Mw = ~10,000 Da), formic acid (88%, Macron Fine Chemicals), and permethrin (CAS # 52645-53-1, PESTANAL^®^, analytical standard) were purchased from Sigma-Aldrich (St. Louis, MO, USA). High Performance Liquid Chromatography (HPLC)-grade methanol (> 99.9%), ammonium sulfate, and glacial acetic acid were purchased from Fisher Scientific (Hampton, NH, USA) and used as received. The nylon 6 warp-knit (tricot) (item # 1410001, ~73 g/m^2^) and nylon 66 double weft-knit (interlock) fabrics (item # 1410003, 260 g/m^2^) were purchased from TestFabrics, Inc. (West Pittston, PA, USA).

### 2.2. Electrospinning

The spinning dopes containing 20 wt % nylon 6 in 88% formic acid with 0 and 2.5 wt % (on the weight of nylon 6) were prepared on a wrist shaker (Burrell Scientific Inc., Pittsburgh, PA, USA) overnight prior to electrospinning. Permethrin was first dissolved in 0.5 mL methanol and then added to the nylon 6/formic acid system in a 20 mL vial for dispersion. As reported in our previous study on electrospinning nylon 6 [24], the parameters during the electrospinning were: a feed rate of 0.5 mL/h (PHS Ultra syringe pump, Harvard Apparatus, Holliston, MA, USA), the applied high voltage of 25 kV (Gamma High Voltage Research Inc., Ormond Beach, FL, USA), a 22-gauge (Inner Diameter: 0.13 mm) needle, a copper sheet wrapped with aluminum as the collector, a tip-to-collector distance of 15 cm (167 kV m^−1^ electric field), and an eight-hour collection time for each nonwoven sample.

### 2.3. Oxygen Plasma Treatment

The plasma treatment method reported by Yip et al. [25] was used to treat the polyamide fabrics. The PE-100 Benchtop Plasma System (Carson City, NV, USA) with a glow discharge generator was employed for the plasma treatment of the samples. Oxygen (O_2_) was chosen as the gas. The discharge power and gas flow rate were 100 W and 50 cm/min, respectively. The exposure time was 10 min. Samples were placed in frames designed from Teflon squares (5 × 10 cm on the outside and 4 × 8 cm cut-out on the inside) cut using a laser cutting machine. These frames served to hold the samples in place during plasma coating, due to the high energy of the incident plasma beam.

### 2.4. Preparation of Insect-Repellent Polyamide Fabrics

Permethrin was applied onto the control untreated and plasma-treated electrospun nylon 6 nonwovens (denoted as ES_N6), commercially available nylon 6 warp-knit tricot (denoted as T_N6), and commercially available nylon 66 double weft-knit interlock (denoted as K_N66) in dosages of 0 and 2.5 % (wt %, on weight of fabrics (o.w.f.) from an aqueous liquor (liquor ratio = 1:30, i.e., 1 gram fabric vs. 30 mL liquid)) that contained 4% (wt %, o.w.f.) (NH_4_)_2_SO_4_; the pH value was adjusted to 6 using acetic acid. The required permethrin was first dissolved in methanol at 1 mg/mL concentration and then added to distilled water with vigorous agitation using a Vortex mixture (Scientific Industries, Inc., Bohemia, NY, USA). The fabrics were then taken out of the solution and a uniform rolling was applied using a padder machine (Jeweler Supermarket, Glendora, CA, USA) until 120 wt% of insecticide solution liquid was added to the fabrics compared to its original weight. The purpose of padding is to ensure a uniform coating across the entire fiber surface. After rolling through the padder, samples were placed in dry petri dishes in the fume hood at room temperature (RT) for 30 min, and further at 80 °C in a vacuum oven after air-drying. The samples were then packed in waterproof and air-tight conditions using aluminum foil and stored in a desiccator under ambient conditions away from UV exposure and protected from the atmosphere. 

### 2.5. Characterization of Polyamide Samples

#### 2.5.1. Surface Morphology

The topographical surface morphology of the nylon 66 Knit (K_N66), nylon 6 tricot (T_N6), and nylon 6 nanofibers (ES_N6) were characterized using the LEO 1550 Keck FESEM. The high-resolution microscopy images provided clear visibility of the microstructure of the fibers. The samples were sputter-coated with gold–palladium for 30 s prior to observation with a LEO 1550 FESEM using a 2 kV accelerating voltage and 50:50 mixture of SE2 and InLens detectors. The average fiber diameter was calculated for each sample using the ImageJ software with three different images of each sample and 50 different measurements from each image. 

#### 2.5.2. Capillary Flow Porometry

An 1100-AEHXL capillary flow porometer (Porous Media Inc., Ithaca, NY, USA) was used in this study to measure the pore size and distribution of as-spun ES_N6, as-received K_N66, and T_N6 polyamide fabrics. Calwick with a defined surface tension of 20.1 dynes/cm (Porous Media Inc., Ithaca, NY, USA) was used as the wetting agent for porometry measurements. The pore size and distribution were calculated by the software using the following equation [26]:

(1) $$D=\frac{4\gamma \;cos\;\theta }{p}$$

where *D* is the pore diameter; *γ* the surface tension of the wetting liquid; *θ* the contact angle of the wetting liquid; and *p* is the differential pressure.

#### 2.5.3. Fourier Transform Infrared (FTIR)

The Fourier transform infrared (FTIR) spectra of permethrin incorporated ES_N6, as-spun ES_N6, as received T_N6, and K_N66, were collected on a FTIR spectrometer (Magna 560, Nicolet Instrument Technologies, Inc., Madison, WI, USA) using the attenuated total reflectance (ATR) mode. The data were recorded in the range of 4000–600 cm^−1^ with a resolution of 4 cm^−1^ and a total of 64 scans for each spectrum.

#### 2.5.4. X-ray Photoelectron Spectroscopy (XPS)

The surface composition of the permethrin-treated polyamide fabrics was evaluated with a Surface Science SSX-100 X-ray photoelectron spectroscope (XPS) (Evans Analytical Group LLC, Sunnyvale, CA, USA). Al Kα X-rays were used as the source for the experiment with a Ni-grating shield present over the samples. Survey, carbon, nitrogen, and oxygen, as well as high-resolution chlorine scans, were used throughout experimentation to provide the surface-state chemical composition for each sample. The carbon and oxygen scans had a pass energy of 50 v, whereas the survey scan was 150 v. Each sample was tilted at a 55 °C take-off-angle (TOA) and precisely aligned with the geometry of the machine. The precise atomic percent (at %) composition of each element present in the sample was evaluated using a curve-fitting program in CasaXPS (v.2.2.12). Chemical bonding of the N, O, C, and Cl present in the sample was then determined based on the interactions between each of the elements.

#### 2.5.5. Tensile Testing

The mechanical properties of the K_N66, T_N6, and ES_N6 polyamide fabrics were evaluated using an Instron 5566 (MA, USA) based on the ASTM Standard Test Method D638-2014 for Tensile Testing Properties of Plastics. A minimum of five sample sets were collected for each fabric with a constant Dogbone width of 3.18 mm, length of 9.53 mm, and varying thickness measured using BeadSmith XL-9030 Metric Digital calipers. A 100 N load and 10 mm/min crosshead speed was used. 

### 2.6. Characterization of Permethrin-Treated Polyamide Fabrics

#### 2.6.1. Determination of Permethrin Concentrations in the Polyamide Fabrics

Gas chromatography–mass spectrometry (GC–MS) characterization was performed using a Hewlett-Packard GC 6890 Series coupled to the Agilent Technologies 5973 N MSD system. The system was equipped with a Hewlett Packard 190915-433 capillary column (30 m × 250 µm i.d., 0.25 µm nominal film thickness). The injection volume was 2 µL, the helium carrier-gas flow rate was 1.25 mL/min, and the split-vent flow rate was 20 mL/min. The oven temperature was programmed to start at 185 °C and hold for 30 min, increase to 220 °C at 10 °C/min, hold at 220 °C for 30 min, increase to 300 °C at 30 °C/min, and hold at 300 °C for 10 min. Mass spectra were recorded at scan range 80–250 m/z, and the threshold for the scan mode was 200. Assignment of possible degradation products was based on the match with standard mass spectrum available in the GC–MS library database [27]. The permethrin content of treated polyamide fabrics was determined by GC–MS analysis after extraction with methanol (1 g fabrics/30 mL methanol; 30 min at 80 °C). The exact concentration was determined by the use of GC–MS and a straight calibration line of pure permethrin. 

#### 2.6.2. Washing Fastness of the Permethrin-Treated Polyamide Fabrics

The electropun nylon 6 incorporated (I-ES_N6) with permethrin during electrospinning, plasma-treated P-ES_N6, P-K_N66, and P-T_N6 were investigated with regard to the fastness of the insecticides during washing at 40 °C for 30 min following the method reported by Kettel et al. [5]. The liquor ratio of the washing was 1:50 (i.e., 1 g of polyamide fabrics in 50 mL of soap solution). The fabrics were then further washed with water for 10 min and two times with distilled water. The evaluation of the insecticide resistance to washing was performed by determination of the permethrin contents on the washed polyamide fabrics by GC–MS analysis after extraction of the polyamide fabrics with methanol at the liquor ratio of 1:30.

#### 2.6.3. Light (UV) Fastness of the Permethrin-Treated Polyamide Fabrics 

The light (ultraviolet, UV) fastness testing of the permethrin-containing polyamide fabrics was conducted using 1 × 6 cm samples from each testing condition. Three 1 × 6 cm strips of each sample were exposed to direct sunlight in a Pyrex petri dish for a duration of four hours. The evaluation of the amount of permethrin on the UV-exposed polyamide fabrics was conducted with GC–MS analysis after extraction of the polyamide fabrics with methanol at the liquor ratio of 1:30.

#### 2.6.4. Assessment of Insect-Repellent Performance

The assessment of mosquito-repellent performance was conducted following the procedure reported in our previous study [28]. Female yellow fever mosquitoes (Aedes aegypti) were used for the study. The slightly modified contact irritancy assay protocol outlined by Grieco et al. [29] was used for assessing the repellency of the various permethrin-treated fabrics. Untreated fabrics were used as control. Permethrin-treated fabric sheets (11.5 cm × 8 cm) were cut to wrap around the inside of the contact irritancy chamber. Three pieces of fabric were draped around the interior of the exposure chamber for each type of fabric. This allowed for the evaluation of the irritancy of the treated fabric toward adult, female mosquitoes. For fabrics that cause a high level of contact irritancy, more mosquitoes will migrate into the untreated, clear “counting” chamber. The number of mosquitoes knocked down in both the exposure chamber and the clear counting chamber was also recorded as a measure of a metric of repellency and insecticidal efficacy. Six replicates were completed for each treatment. Control treatments of untreated fabrics were tested in parallel with each permethrin-treated type of fabric.

## 3. Results and Discussion

### 3.1. Morphology of Polyamide Fabrics

**Figure 1:**
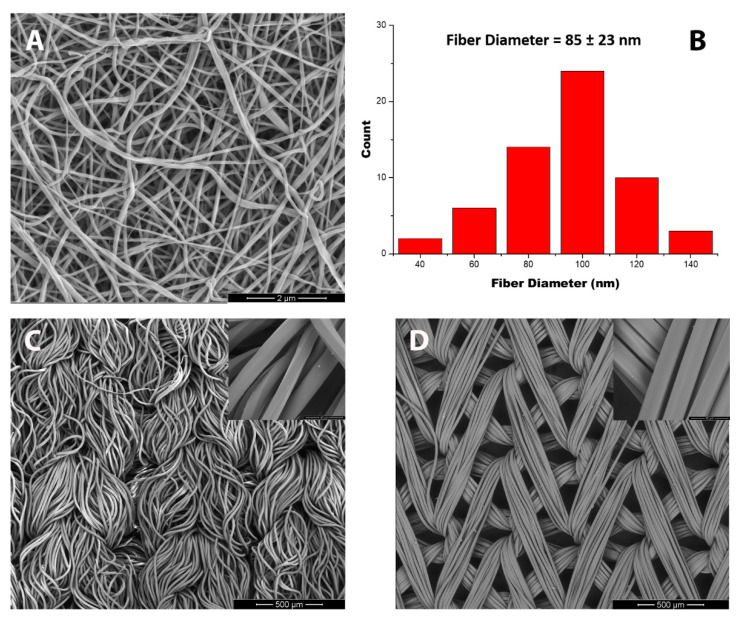
FE-SEM imaging of (**A**) nylon 6 nanofibers (ES_N6); (**B**) Fiber diameter distribution of ES_N6, (**C**) nylon 66 knit (K_N66), and (**D**) nylon 6 tricot (T_N6).

Figure 1 shows the structural properties of nylon 6 nanofibers (ES_N6) (Figure 1A), nylon 66 Knit (K_N66) (Figure 1C), and nylon 6 tricot (T_N6) (Figure 1D) polyamide fabrics. ES_N6 is an electrospun nonwoven fabric in which the fibers are randomly packed. The average fiber diameter of ES_N6 is 85 ± 23 nm. The fiber diameter distribution of the ES_N6 is shown in Figure 1B. K_N66 is a commercially available double weft-knit interlock with an average fiber diameter of 16 ± 1 um. T_N6 is a commercially available warp-knit tricot with an average fiber diameter of 21 ± 1 um. Both K_N66 and T_N6 show uniform fiber diameter. These properties of the polyamide fabrics are particularly significant, because the surface area-to-weight ratio is crucial for the application of insecticide to the surface of the fabrics. The exponentially larger surface area/mass ratio of the electrospun nanofiber fabrics (ES_N6) favors the insecticide absorption. 

### 3.2. Pore Size and Distribution of Polyamide Fabrics

**Figure 2:**
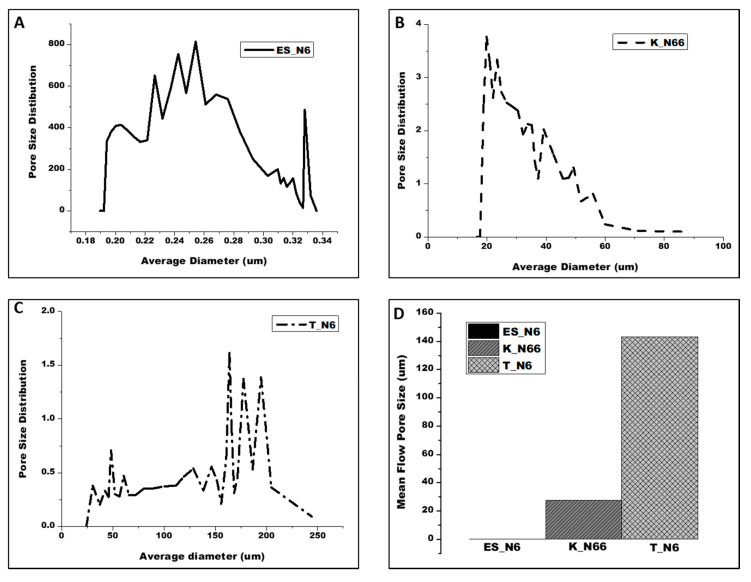
Pore size distribution and mean flow pore size of: (**A**) nylon 6 nanofibers (ES_N6); (**B**) nylon 66 knit (K_N66); (**C**) nylon 6 tricot (T-N6); (**D**) comparison of the mean flow pore size of ES_N6, K_N66, and T_N6.

Capillary flow porometry testing provides the average pore size and pore size distribution for each polyamide sample. Figure 2 shows the pore size distribution of the polyamide samples. Table 1 shows the mean flow pore size of the polyamide samples. T_N6 samples showed the largest average pore size of 143.2 um, followed by K_N66 samples with 27.6 um, and finally ES_N6 samples with 0.23 um. The average pore size of the electrospun nylon 6 (ES_N6) is about 620% less than that of nylon 6 tricot (T_N6). 

### 3.3. FTIR Spectra of As-Spun/Received Polyamide Fabrics

**Figure 3:**
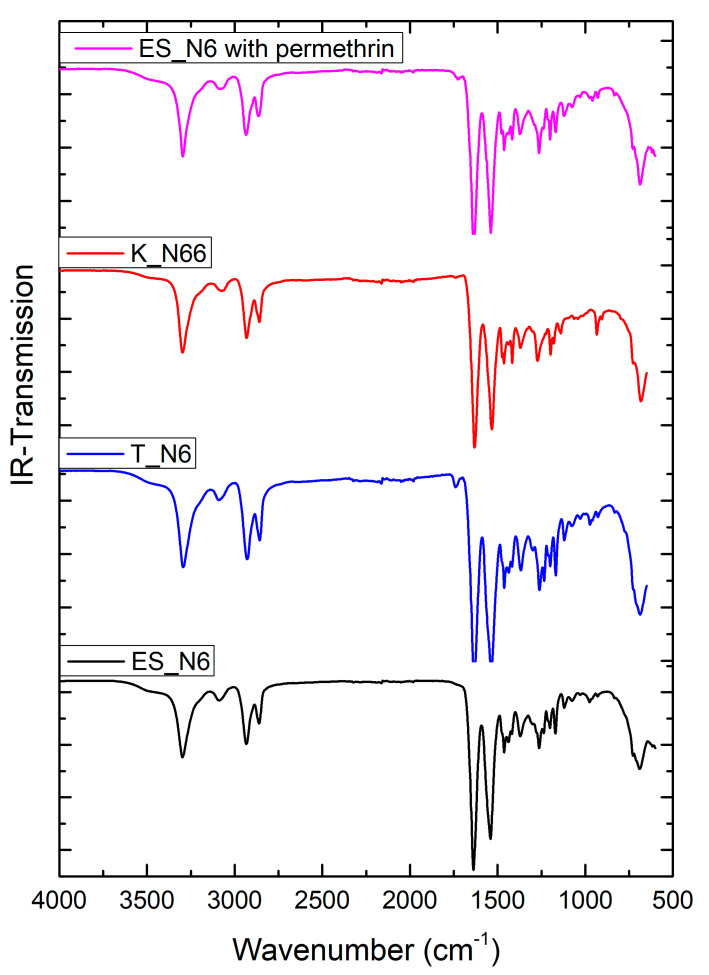
FTIR spectra of polyamide samples.

Figure 3 shows the FTIR spectra of electrospun nylon 6 incorporated with permethrin during electrospinning, as-spun ES_N6, as-received K_N66, and T_N6 polyamide samples. FTIR evidenced C=O bond stretching at around 1640 cm^−1^ and N-H bond bending around 1540 cm^−1^. These peaks provide a strong indication that polyamides are present in the sample and elicit a confidence in confirming the presence of nanofiber polyamide fabrics as spun from the 20 wt% Nylon-6 in 88% formic acid spinning dope. No permethrin characteristic peaks were observed in the ES_N6 with permethrin samples; one possible reason for this is that the amount of permethrin was too low to be detected by AR. 

### 3.4. XPS Results of Plasma-Treated and Untreated Polyamide Samples

**Figure 4:**
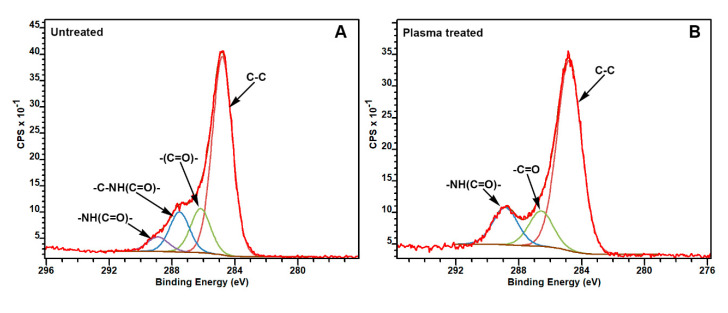
High-resolution XPS spectra of the C1s binding energy region of polyamide fabrics before (**A**) and after plasma treatment (**B**). The positions of different functional groups are indicated.

X-ray Photoelectron Spectroscopy was employed to identify the polar groups attached after the plasma treatment. C1s core-level spectra of polyamide-untreated (Figure 4A) and plasma-treated (Figure 4B) samples were deconvoluted with four and three components, respectively. In terms of binding energy, the peak at 284.80 eV is attributed to C in the C–C chain CH2 groups. The peak at 286.10 eV can be associated to the amido-carbonyls [–(C=O)]. The peak at 287.40 eV represents the carbon atoms neighboring the amido nitrogen [–C–NH(C=O)–], and that of 289.03 is assigned to the amide carbonyl carbons [–NH(C=O)–]. An increase in the peak at 289.03 was observed, which can be attributed to a low-level oxidation of the methylene carbons promoted by the plasma treatment [11]. 

The oxidation was also confirmed by the increase in the atomic concentration of oxygen on the surface (shown in Table 2). After the plasma treatment, the atomic concentration of oxygen on the surface of ES_N6, K_N66, and T_N6 increased by 31%, 30%, and 16%, respectively. The O1s/C1s ratio increased from 0.192 for the untreated to 0.293 for the plasma-treated samples. It is suggested that the plasma treatment induced the formation of carboxylic species on the surface, either in the hydrocarbon or carbonyl groups, which finally enhances the hydrophilicity of the polymer [12]. The oxygen uptake can be attributed to the presence of atomic oxygen during the process, resulting from the dissociation of atmospheric O_2_ and the reaction of the resulting “active” surface obtained after the plasma modification [12,30]. It is known that the plasma treatment is responsible for chain scission on the polymer surface and, thus, can react with the environment prior to reaching equilibrium.

### 3.5. Tensile Properties of Polyamide Fabrics

**Figure 5:**
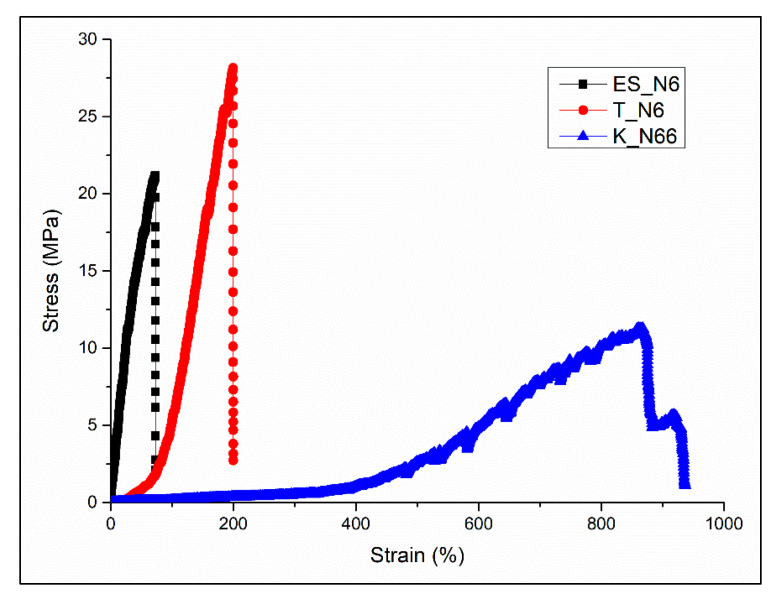
Typical stress–strain curves of polyamide fabrics: ES_N6, T_N6, and K_N66.

Figure 5 shows the typical stress versus strain plots of ES_N6, K_N66, and T_N6 polyamide fabrics. As confirmed by the values presented in Table 3, the ES_N6 and T_N6 fabrics showed no significant (*p* < 0.05) difference in their tensile strength and Young’s modulus. The highly packed nanofibers in the ES_N6 nonwoven fabrics in which fibers are randomly aligned without any interloping, as in knits, contributed the force sharing during tensile deformation and, hence, demonstrated similar tensile strength compared with the warp-knit tricot (T_N6), in which the fiber diameter is approximately 250 times bigger in fiber diameter. The electrospun nylon 6 samples (ES_N6) demonstrated high industry feasibility in the development of protective clothing with good mechanical properties for daily wear. The double weft-knit interlock (K_N66) showed significantly (*p* < 0.05) larger elongation due to the interlooping of the yarns in the double weft-knit fabrics. 

### 3.6. Permethrin Contents on Polyamide Samples

**Figure 6:**
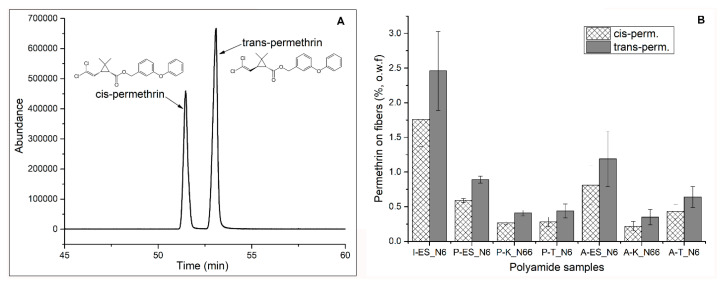
(**A**): GC chromatogram of cis- and trans-permethrin, and (**B**): Permethrin (Perm.) content (wt%, o.w.f., trans- and cis-permethrin) on polyamide fabrics after application (I—incorporation during fiber formation, P—plasma-treated, and A—as-spun/received, untreated samples).

Figure 6 shows the permethrin contents (based on the weight of fabrics, o.w.f.) applied to the polyamide fabrics by dip coating on the surfaces and incorporation into fibers during the electrospinning. Figure 6A is the Gas Chromatography (GC) chromatogram of cis- and trans-permethrin. Figure 6B is the permethrin contents on different polyamide samples: the electrospun nylon 6 with incorporation (I-ES_N6) of permethrin, plasma-treated samples (P-ES_N6, P-K_N66, and P-T_N6), and as-spun/received samples (A-ES_N6, P-K_N66, and A-T_N6) with dip coating permethrin. Table 4 shows the permethrin wet pickup efficiencies on the polyamide samples. The wet pickup efficiencies were calculated by comparing the permethrin contents (obtained from GC analysis) on the fabrics to the theoretical amounts of permethrin added to the polymer solutions for electrospinning and the dispersions for dip coating, which was 2.5 wt% based on the weight of polymer/fabrics. The incorporation of permethrin into the polymer solution before the formation of fibers demonstrated the most efficient way of applying the active ingredient to the fiber/fabric systems. Approximately 98% permethrin was incorporated nylon 6 fibers during the fiber formation. The plasma treatment did not improve the overall amount of permethrin applied to the polyamide fabrics compared to the untreated (as-spun) counterpart samples. 

**Figure 7:**
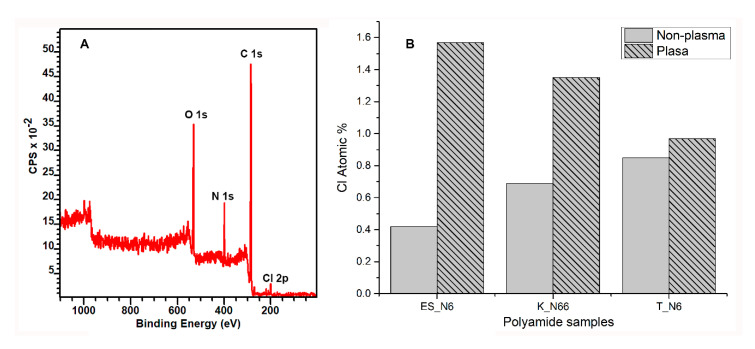
XPS confirmation of the application of permethrin on the polyamide fabrics. (**A**): Survey scan graph of the polyamide samples with permethrin collected at 55° take off angle (**B**): Cl atomic percentage comparison of the untreated and plasma-treated polyamide fabrics.

The presence of the permethrin on the surface of polyamide fabrics was further confirmed by XPS analysis. The survey scan XPS spectrum (Figure 7A) of the polyamide fabric with permethrin shows the photoelectron lines at the binding energies of about 200.61, 284.86, 399.61, and 531.86 eV contributing to Cl 2p, N 1s, and O 1s, respectively. The Cl 2p indicates the existence of permethrin on the surface of the polyamide fabrics. Figure 7B shows a different Cl atomic percentage on the untreated and plasma-treated polyamide fabrics by dip coating. Plasma-treated ES_N6 and K_N66 showed a significant increase in the permethrin uptake compared to their untreated counterparts. The amount of permethrin on the surface of the plasma-treated polyamide fabrics significantly increased compared to the untreated samples, while the T-N6 sample did not demonstrate significant difference. The untreated and plasma-treated ES_N6 samples showed the most significant difference in permethrin uptake after dip coating among the three types of polyamide fabrics. The high surface area of the electrospun nylon 6 nanofiber nonwoven fabrics favors the resulting “active” surface obtained after the plasma treatment. The significantly small pore size, narrow pore distribution, and high surface area, evident from both the FESEM and porometry data, solidify the enhanced ability of nanofibers to uptake permethrin from the insecticide solution to coat a larger surface area of fibers.

### 3.7. Washing/Light Fastness of Permethrin-Containing Polyamide Fabrics

**Figure 8:**
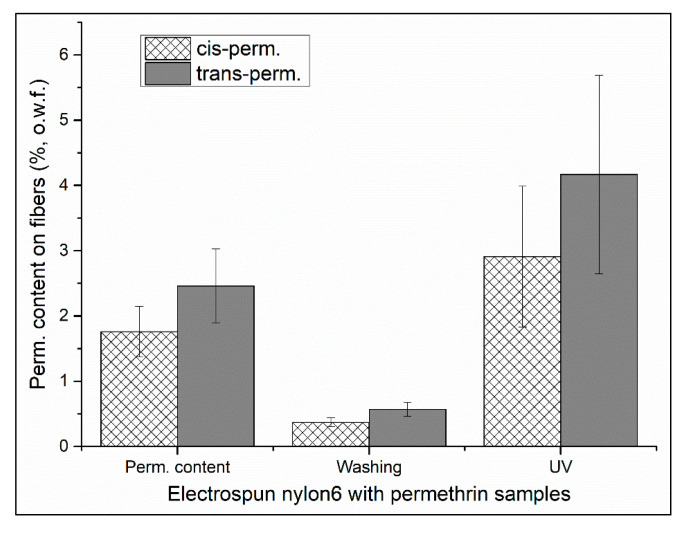
Permethrin contents on electrospun nylon 6 fibers incorporated with 2.5% (o.w.f.) permethrin after formation, after a washing process and after four hours of direct sunlight exposure.

The electrospun nylon 6 with 2.5 wt % permethrin incorporated to the solutions before the formation of the fibers was investigated with regard to fastness of insecticides during washing or to the light. Figure 8 shows the permethrin contents (in wt %, o.w.f., cis- and trans-permethrin) on the I-ES_N6 fabrics after formation, after a washing process and after four hours of direct sunlight exposure. The permethrin content on the nanofibers decreased dramatically (approximately 78% weight loss) after washing; a possible reason for this was because of the insolubility of permethrin in the nylon/formic acid solution, which resulted in poor physical bonding between the permethrin and the fibers. Four-hour direct sunlight exposure did not result in a significant change in the permethrin content on the I-ES_N6. Further study needs to be done by following a standard testing method, such as DIN EN ISO 105-B02. 

**Figure 9:**
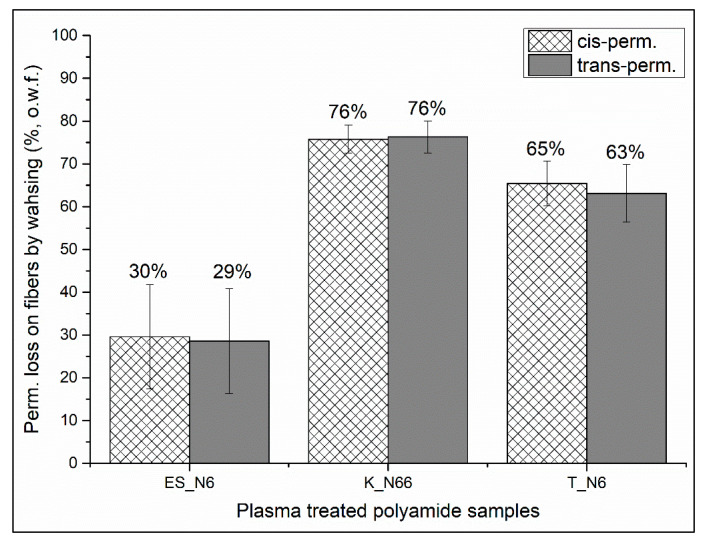
Weight loss of permethrin on plasma-treated polyamide fabrics after washing.

The washing test of the plasma-treated polyamide fabrics (ES_N6, K_N66, and T_N6) is shown in Figure 9. After one washing process, about 30%, 76%, and 63% of the cis- and trans-permethrin are removed from the plasma-treated electrospun, double weft-knit interlock, and warp-knit tricot polyamide fabrics. The plasma-treated electrospun nylon 6 demonstrated the best fastness to washing, due to the significantly small pore size, narrow pore distribution, and high surface area. No significant difference was observed from the two commercially available polyamide fabrics.

### 3.8. Contact Irritancy Assay of Mosquito Repellency

**Figure 10:**
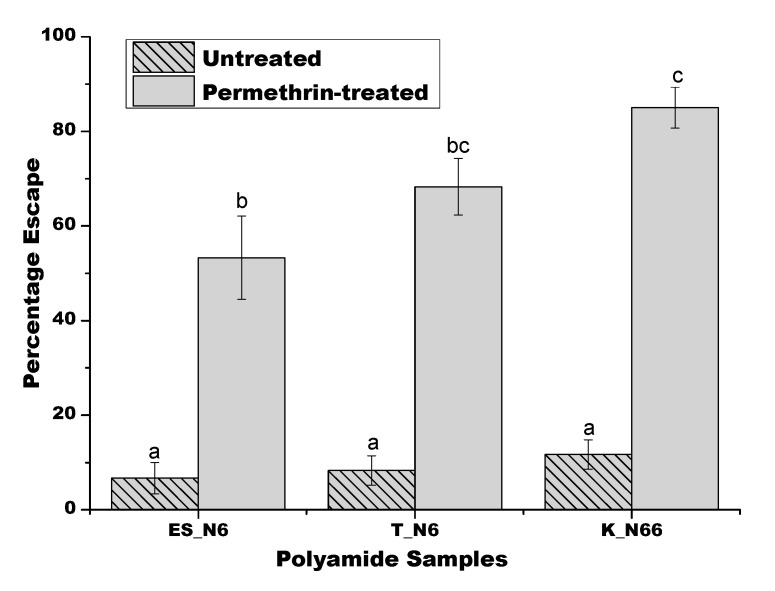
Contact irritancy of various untreated and permethrin-treated fabrics against adult female *Aedes aegypti* after a five-minute exposure interval. The differences in efficacy are described by the letters above, with each bar highlighting statistically significant comparisons (one-way ANOVA with a post-hoc Bonferroni t-test).

The assessment of contact irritancy, a measure of contact repellency, was successful in the five-minute time interval. Control untreated fabrics produced a sufficiently lower escape percentage compared to the permethrin-treated fabrics to allow for the assessment of repellency. Mosquitoes responded well in each of the experimental intervals, and the escape frequencies for each tested fabric were normally distributed and consistent. No outliers in mosquito response were observed. The treatment effect (i.e., type of fabric screened) was statistically significant in the one-way ANOVA model used to assess statistical significance with an F value of 45.88 with five degrees of freedom (*p* < 0.001). This allowed for further comparisons between treatments using a post-hoc Bonferroni-corrected t-test. After a five-minute exposure of mosquitoes to each fabric, the responses in escape frequencies were 6.7 ± 3.3%, 8.3 ± 3.1%, and 11.7 ± 3.1% of mosquitoes migrating into the clear viewing chamber for untreated ES_N6, T_N6, and K_N66, respectively (Figure 10). The escape frequencies of the control untreated fabrics were statistically similar to each other, but statistically lower compared to the permethrin-treated fabric. 

Among the permethrin-treated fabrics, there were differences observed in the escape frequencies of exposed mosquitoes at five-minute exposure time intervals. The escape frequencies ranged from 53.3 ± 8.8% to 85 ± 4.3% for the permethrin-treated ES_N6 and K_N66 fabrics, respectively. The permethrin-treated T_N6 caused an escape frequency of 68.3 ± 6%, and it was not statistically distinct compared to permethrin-treated fabrics of both the ES_N6 and T_N6 fabrics. At this exposure time interval, permethrin-treated K_N66 fabric caused the highest level of contact irritancy compared to the other two permethrin-treated fabrics. The K_N66 fabrics performed similarly to permethrin-treated T_N6, and caused a significantly higher level of contact irritancy than the permethrin-treated ES_N6. All permethrin-treated fabrics caused a higher percentage escape of mosquitoes from the exposure chamber than the corresponding untreated fabrics. Permethrin-treated K_N66 produced a higher percentage of exposed mosquitoes to escape compared to the permethrin-treated ES_N6. Permethrin-treated T_N6 caused a similar percentage to escape compared to both other permethrin-treated fabrics.

Table 5 shows the sublethal effects observed in mosquitoes exposed to the permethrin-treated fabrics for five minutes. Both the permethrin-treated versions of ES_NS and T_N6 fabrics did not produce significant knockdown in the viewing chambers or the exposure chambers. Permethrin-treated K_N66 produced significant knockdown in the viewing chamber compared to the other permethrin-treated fabrics. It is apparent that mosquitoes exposed to some of these permethrin-treated fabrics began to experience toxic effects. The primary signs of exposure toxicity included knockdown in the viewing chamber and/or the exposure chamber. Knockdown in the viewing chamber can be indicative of latent toxicity of the repellent after the mosquito has escaped into the repellency chamber. This is indicative of a repellent that produces a rapid excitatory response in the mosquitoes after coming into contact with the treated-surface, followed by a toxic effect after the mosquito is no longer exposed in the viewing chamber. This is common for mosquitoes exposed to permethrin, an excito-repellent, at sufficiently repellent levels [31]. Knockdown in the exposure chamber may indicate that significant toxicity is occurring immediately following exposure to the repellent. This may be due to the intrinsic toxicity of the repellent compound, the concentration at which it is applied to the treated surface, or the exposure time interval. Significant knockdown in the exposure chamber may indicate that mosquitoes are unable to move freely into the viewing chamber and may decrease the escape frequency. Significant sublethal effects may indicate that mosquitoes may not be able to freely move between chambers. Permethrin-treated K_N66 produced low levels of knockdown in the viewing chamber. The other permethrin-treated fabric produced little-to-no knockdown effects.

## 4. Conclusions

The incorporation of permethrin into the nylon 6 solution before the formation of nanofibers demonstrated the most efficient way to apply active ingredients to the fiber/fabric systems. The high surface area of the electrospun nylon 6 favors the resulting “active” surface obtained after the plasma treatment. The significantly small pore size, narrow pore distribution, and high surface area solidified the enhanced ability of nanofibers to uptake permethrin from the insecticide solution to coat a larger surface area of fibers. The plasma treatment did not improve the overall amount of permethrin uptake on the polyamide fabrics. However, the plasma treatment significantly increased the amount of permethrin on the surface of the fabrics. All permethrin-containing polyamide fabrics showed excellent fastness of the insecticide to light. The electrospun nylon 6 nonwovens demonstrated the best fastness to washing among the plasma-treated, electrospun nylon 6, nylon 66 double weft-knit interlock, and nylon 6 warp-knit tricot. The assessment of contact irritancy demonstrated that the control untreated fabrics produced a sufficiently lower escape percentage compared to the permethrin-treated fabrics to allow for the assessment of repellency. All permethrin-treated fabrics were observed to be repellent, with higher escape frequencies that were statistically significant compared to each corresponding control untreated fabric.

## Figures and Tables

**Table 1:** Mean flow pore size of polyamide fabrics.

Samples	Mean Flow Pore Diameter (um)
ES_N6	0.23 ± 0.05
K_N66	27.6 ± 1.0
T_N6	143.2 ± 0.2

**Table 2:** XPS results of plasma-untreated and treated samples.

Polyamide Samples	O1s/C1s		O1s Increasing after Plasma (%)
	Untreated	Plasma	
ES_N6	0.192	0.263	31
K_N66	0.216	0.309	30
T_N6	0.897	0.998	16

**Table 3:** Summary of the tensile properties of the polyamide fabrics: ES_N6, T_N6, and K_N66.

Samples	Ultimate Tensile Strength (MPa)	Elongation at Failure (%)	Young’s Modulus (MPa)
K_N66	6.12 ^a^	898.8 ^c^	1.87 ^f^
T_N6	25.65 ^b^	252.84 ^d^	21.03 ^g^
ES_N6	17.4 ^b^	74.85 ^e^	32.48 ^g^

Note: Different letters represent significantly different values (*p* < 0.05).

**Table 4:** Wet pickup efficiencies of permethrin on the polyamide fabrics. (nylon 6 nanofibers (ES_N6), nylon 66 Knit (K_N66), nylon 6 tricot (T_N6), I—incorporation permethrin during fiber formation, P—plasma-treated, and A—as-spun/received, untreated samples).

Samples	Capture Efficiencies (%)
I-ES_N6	98.4 ± 22.8
P-ES_N6	35.6 ± 2
P-K_N66	16.4 ± 1.6
P-T_N6	17.6 ± 4
A-ES_N6	47.6 ± 16
A-K_N66	14 ± 4.4
A-T_N6	25.6 ± 6

**Table 5:** Sublethal effects other than repellency caused by various permethrin-treated fabrics after a five-minute exposure interval.

Permethrin-Treated Fabrics	Knockdown in Viewing Chamber	Knockdown in Exposure Chamber
ES_N6	1.67 ± 1.67	0 ± 0
T_N6	5 ± 3.42	0 ± 0
K_N66	20 ± 4.47	1.67 ± 1.67
